# Impact of childhood trauma on functioning of patients with bipolar disorder

**DOI:** 10.1192/j.eurpsy.2022.923

**Published:** 2022-09-01

**Authors:** D. Bougacha, S. Ellouze, R. Jenhani, R. Ghachem

**Affiliations:** 1 Razi hospital, B, Manouba, Tunisia; 2 Hedi Chaker University Hospital, Psychiatry B, Sfax, Tunisia

**Keywords:** Childhood Trauma, functioning, bipolar disorder

## Abstract

**Introduction:**

Exposure to severe childhood trauma has been associated with the onset and the severity of bipolar disorder in adults.

**Objectives:**

The aim of this study was to examine the relationship between childhood trauma and functioning of patients suffering from bipolar disorder.

**Methods:**

We conducted a cross-sectional, descriptive, and analytical study, including sixty-one remitted patients with BD. We used the Childhood Trauma Questionnaire (CTQ-SF) to measure history of traumatic childhood experiences and the Functioning Assessment Short Test (FAST) to assess functioning.

**Results:**

The mean age of patients was 43.4. The sex ratio was 2.4. Almost two-thirds of patients (64%) had experienced at least one type of childhood trauma. An overall functional impairment was found in 70.5% of participants. The CTQ total score was significantly associated with low educational level (p=0.001), low socio-economic status (P=0.034), a family history of psychosis (P=0.022), the number of mood episodes (P=0.001), the number of hospitalizations (P=0.04), the number of relapses with psychotic features (p=0.002) and that of depressive relapses (P<0.001), rapid cycling (P=0.012), higher rates of suicide attempts (P=0.04) and poor functioning (P<0.001).The logistic regression analyses showed a significant association of childhood trauma with low educational level (p=0.001), high number of depressive episodes (p=0.013) and poor functioning (p<0.001).

**Conclusions:**

Our findings demonstrate that childhood abuse and neglect are risk factors associated with worsening clinical course of bipolar disorder and higher functional impairment. These findings press the urgency for preventive practices and early intervention strategies to diminish the prevalence of childhood trauma and minimize their impact.

**Disclosure:**

No significant relationships.

